# Assessment of early neonatal practices to prevent hypothermia ; A comparative study

**DOI:** 10.12688/f1000research.154628.1

**Published:** 2024-08-20

**Authors:** Smrithi GM, Gayathri Renganathan, Rohith Motappa, Nitin Joseph, Ravikiran SR

**Affiliations:** 1 Kasturba Medical College Mangalore, Manipal Academy of Higher Education, Karnataka, Manipal, 576 104, India; 2 Senior Resident, Department of Paediatrics, Kasturba Medical College, Mangalore, Manipal Academy of Higher Education, Karnataka, Manipal, 576 104, India; 3 Assistant Professor, Department of Community Medicine, ESIC Medical College & PGIMSR and Model Hospital, Rajajinagar Bangalore-560010, Karnataka, India; 4 Associate Professor, Department of Community Medicine, Kasturba Medical College Mangalore, Manipal Academy of Higher Education, Karnataka, Manipal, 576 104, India; 5 Professor, Department of Paediatrics, Kasturba Medical College Mangalore, Manipal Academy of Higher Education, Karnataka, Manipal, 576 104, India

**Keywords:** Neonatal Hypothermia, Prevention, Video based education, Knowledge, Practice

## Abstract

**Background:**

Globally, neonatal deaths are significantly attributed to hypothermia. This is mostly because of its co-morbidity with asphyxia, premature birth and severe neonatal infections. Worldwide, neonatal hypothermia case fatality rates (CFRs) range from 8.5% to 52%. This study aimed to assess how well a video-based training intervention on mothers’ knowledge and practices in preventing neonatal hypothermia worked.

**Methods:**

The knowledge and practices of two groups of primi-para mothers—one control group and one intervention group—were compared in order to achieve this. A total of 124 primipara mothers took part in the research. Using a video based tool, the intervention group was educated about preventing hypothermia in newborns. Both control and intervention group mothers were interviewed to compare the knowledge and practices.

**Results:**

Sixty-one of the sixty-two mothers in the control group were unsure of which body area to cover in order to preserve the baby’s body heat. Following the intervention, 61 out of 62 mothers in the group recognised which body part to cover to protect the baby’s body heat. In the post-intervention group, 98.4% of moms wore a cap over their baby’s head, compared to just 35.5% in the control group.

**Conclusion:**

The results of this study demonstrate the significant improvement in mothers’ knowledge and actions about the prevention of neonatal hypothermia resulting from the use of a video-based training tool.

## Introduction

A core body temperature of less than 36.5 °C or a skin temperature of less than 36 °C is considered neonatal hypothermia, according to the World Health Organisation (WHO). ^1^ A baby is said to have moderate hypothermia if their temperature is between 32.0 and 34.9°C (89.6 and 96.6°F), and severe hypothermia if it is lower. ^2^ Hypothermia is a significant contributor to newborn fatalities globally, even though it seldom ends in death. This is mainly due to its co-morbidity with severe neonatal infections, premature birth, and asphyxia. ^3^ Hypothermia engenders a vicious cycle with physiological abnormalities such as hypoxia, hypoglycemia and shock. ^4^

Every year, an estimated four million newborns die. ^5^ Over 99 percent of newborn fatalities take place in underdeveloped nations. One million babies perish in the first month of birth each year, with India alone bearing one-fourth of the burden. ^6^ The case fatality rates (CFR) for newborn hypothermia vary from 8.5% to 52% globally. ^3^ According to a study by Wake et al, neonates with hypothermia had nearly five times the risk of dying as newborns than those with a normal body temperature. ^6^ This study will help achieve the Sustainable Development Goal of the UN, which is to guarantee healthy lives and promote well-being for everyone at all ages. ^7^

Teaching moms how to recognise the symptoms of newborn hypothermia and how to prevent it can be a crucial preventive measure. Only during their hospital stay do newborns have medical supervision. Therefore, it is essential to educate mothers who constantly keep a watch on and tend to the newborn. Researching the effects of maternal education will help reduce newborn hypothermia in general.

## Summary of evidence

Anurag Srivastava et al. carried out a cross-sectional study among new mothers in Western Uttar Pradesh to ascertain the knowledge and practice gaps on newborn thermal care. It was shown that 60.9% of those surveyed knew exactly how to keep a newborn warm after delivery. ^8^

Mahvish Qazi et al. did a study in Northern India on mothers’ awareness of how to prevent neonatal hypothermia. It was shown that while 45% of moms knew how to rapidly wipe a newborn to keep them warm, just 3% of mothers were familiar with kangaroo mother care. ^9^

In Jimma, South West Ethiopia, a research by Kebede et al. indicated that 32.5% of moms practiced early newborn bathing. Since it causes newborn hypothermia and related problems, this needs to be addressed first. ^10^

Nearly half of mothers lack sufficient knowledge about preventing hypothermia, according to a cross-sectional study on the topic conducted by Indu Shah et al. among 70 mothers of newborns. ^11^

In the SNCU of M.K.C.G Medical College Hospital, 54 mothers of low birth weight neonates participated in a study by Sadhana Panda et al. on their understanding of preventing newborn hypothermia. Just 44% of mothers believed that drying the infant right after would stop heat loss, and only 11% of mothers knew enough about skin-to-skin contact, the most effective way to stop heat loss. ^12^

According to a study by Ahmed et al. in three hospitals in the state of Khartoum, only 52.66% of mothers were in favour of treating newborn hypothermia symptoms as soon as they appeared. ^13^

A research by Kibaru et al. in the Nakuru Central district of Kenya found that just 9.7% of mothers recognised hypothermia as a potential danger sign for their newborn. ^14^ According to a research conducted in rural Bangladesh by Zaman et al., hypothermia was the infant risk indication that newly delivered women (RDW) identified the least often(26.1%). ^15^

Smita Srivastava et al.’s randomised control trial found that skin-to-skin contact during the initial postpartum period is associated with improved neonatal temperature regulation. ^16^ Focused interventions reduced the average monthly risk of hypothermia among low birth weight and late preterm neonates to 13.3%, according to research by Christine Andrews et al. Prior to this, the average monthly rate of hypothermia was 29.8%. ^17^

Infants who did not receive their mothers’ first skin-to-skin contact during delivery had a 4.3-fold increased risk of developing hypothermia, as per research by Demissie et al. ^18^

To determine how well kangaroo mother care (KMC) works to keep low birth weight babies warm while being transported from the hospital to their families, Nimbalkar et al. undertook a study. After being discharged from the hospital, the study group experienced a mean rise in temperature of 0.01°C, whereas the control group saw an average decrease in temperature of 0.07°C during transportation. ^19^

As part of the intervention plan for their study, Noel Joseph et al. added a number of interventions, such as key cards, hypothermia algorithms, and nursing education. Following implementation, the mother-baby unit’s incidence of hypothermia dramatically decreased, falling from 20.9% to 14.5%. Furthermore, there were 71% fewer newborns who required admission to the Neonatal Intensive Care Unit (NICU). ^20^

Mukunya et al.’s multivariable analysis revealed that delayed breastfeeding initiation, low birth weight and home birth were the variables linked to a higher risk of newborn hypothermia. ^21^

According to a study by Olawuyi et al., nearly two thirds (66.7%) of mothers in newborn intensive care units were aware of the advantages of Kangaroo Mother Care (KMC). ^22^

In a research by S P Choudhary et al., which involved medical and paramedical personnel, only 47.8% of the participants correctly defined newborn hypothermia, and a noteworthy 52.2% thought it was an uncommon problem. ^23^

In the early postpartum period, when neonates are most susceptible to hypothermia, the warm chain was not continuously maintained, according to a study by Karsten Lunze et al done in rural Zambia. ^24^

Within the first five minutes of birth, 27% of newborns were wrapped and 42% of neonates were dried, according to a study conducted in rural southern Tanzania by Donat Shamba et al. The fact that carers neglected to tend to the baby until after the placenta was delivered was the main cause of the delay in wrapping and drying. ^25^

Early newborn washing was practiced by 87.5% of women who did not visit health facilities for ANC, compared to 54.8% of moms who had four or more ANC visits, according to Gebrew Getachew et al. ^26^

The results of a study by Luke Mullany et al showed that infants whose breastfeeding was delayed for longer than 24 hours were almost 50% more likely to experience hypothermia. ^27^ According to a research by Meseret Ekubay et al, 58.3% of mothers started breastfeeding their newborns in the first hour following birth. ^28^

According to Golicha Wako et al., out of 388 mothers in South Ethiopia, 69.1% of them covered their newborns’ heads right away after delivery, and almost all of them did so for duration of the neonatal period. ^29^

Dongre et al undertook a study to determine how well-informed mothers in periurban Wardha were about infant warning signs. Just 2.8% of respondents identified hypothermia in neonates as one of the warning indicators. ^30^

## Contribution to knowledge

**Methods**

a. **Study setting:** Postnatal wards of Government Lady Goschen Hospital, Mangalore.
b. **Study design:** Non Randomized educational Trial.
c. **Study participants:** Primipara mothers of newborns admitted in the Postnatal wards of Government Lady Goschen Hospital, Mangalore.
  Group A – control group
  Group B **–** received counselling on preventing neonatal hypothermia via a video-based educational tool
d. **Inclusion criteria:**
  Primipara Mothers of newborns in the postnatal ward.
e. **Exclusion criteria:**
  i. Mothers of severely ill newborns at the time of data collection.
  ii. Mothers of babies admitted in NICU.
  iii. Mothers of babies delivered at home.
  iv. Inability of mother to participate. Eg: Mother is severely ill at the time of study.
  v. Mothers who are not willing to participate.
f. **Study duration:** 2 months.
g. **Sample size:** 62 primipara mothers of newborns in the control group and 62 primipara mothers of newborns in the intervention group make up the study’s sample size. Using an article based on new born thermal care as reference, the sample size was determined. ^8^
$$n=\frac{{\left(\;Z\;\alpha \;\right)}^{2}\;\mathrm{pq}\;}{d^{2}}$$
h. **Tool for data collection:** A structured questionnaire about preventing hypothermia in neonates.
i. **Data collection methodology**:
  i. From the postnatal wards, participants were chosen. As the control group, Group A did not get any intervention. Using a video-based intervention, Group B was educated on preventing hypothermia in newborns. Group A mothers were recruited between June 24, 2023 and July 24, 2023 and data was gathered. Group B were enlisted and instructed using the video-based tool the following month. Participants’ questions on preventing newborn hypothermia were addressed. The data collection tool was the same questionnaire as used for Group A. Following a review of literature and expert advice, the semi-structured questionnaire was developed. Validation of the content was completed. The principal investigator conducted interviews with the mothers in order to collect data.
  ii. The principal investigator conducted interviews with both Group A and Group B. Group B was interviewed twenty-four hours after they were educated about preventing neonatal hypothermia. Informed written consent for participation in the study was obtained.
j. **Data analysis**:
  Data was entered in Microsoft Excel 2016 (Microsoft Excel, RRID:SCR_016137) and analyzed using SPSS version 25 (IBM SPSS Statistics, RRID:SCR_019096). Data was interpreted in proportions and percentages, and Chi-square and P-values were obtained.

**Ethical considerations:** The Institutional Ethics Committee at Kasturba Medical College in Mangalore has granted ethical clearance on 16/03/23 (Protocol No: IECKMCMLR-03/2023/94). We have received approval from the Government Lady Goschen Hospital’s Medical Superintendent to carry out this research.

## Results

In our study there were a total of 124 participants, out of which 50% belonged to the intervention group.

Table 1 depicts the demographic information of study participants which implies that out of 62 mothers in the control group, 5 of them were between the ages of 15-20 years (0.08%), 29 were between 21-25 years of age (46.7%), 19 were between 26-30 years of age (30.6%) and 9 were between 31-35 years of age (14.5%). 51 participants had number of family members ranging between 1-5 (82.3%) and 11 participants had between 6-10 (17.7%). Majority of them had studied between 1 ^st^ – 10 ^th^ std (46.8%) and had a family income of Rs.5000-10000 (67.7%) in the control group. Majority of them had a normal vaginal delivery (58.1%) and delivered a term baby (96.8%). Majority of the newborns in the control group were females (56.5%). Out of the 62 newborns in the control group, 6 had a birth weight ranging between 1-1.9 kg (9.7%), 30 between 2-2.9 kg (48.4%) and 26 between 3-3.9 kg (41.9%).

**Table 1.** Demographic characteristics.

Sl. number	Demographical characteristic	Response	Non-Intervention group	Intervention group	Total	P*
1.	Age of mother, years Mean (IQR)*	N	62	62	124	.607
2.	Maternal education	Illiterate	1	0	1	.159
			1.6%	0.0%	0.8%	
		1 ^st^ – 10 ^th^ std	29	23	52	
			46.8%	37.1%	41.9%	
		Higher secondary	15	26	41	
			24.2%	41.9%	33.1%	
		Graduate and above	17	13	30	
			27.4%	21.0%	24.2%	
3.	Type of delivery	Normal Vaginal	36	21	57	.007
			58.1%	33.9%	46.0%	
		Cesarean Section	26	41	67	
			41.9%	66.1%	54.0%	
4.	Family income	Less than 5000 rs	1	0	1	.012
			1.6%	0.0%	0.8%	
		5000-10000 rs	42	26	68	
			67.7%	41.9%	54.8%	
		10000-15000 rs	19	34	53	
			30.6%	54.8%	42.7%	
		More than 15000 rs	0	2	2	
			0.0%	3.2%	1.6%	
5.	Number of members in the family*	N	62	62	124	.094
6.	Sex of the child	Male	27	40	67	.019
			43.5%	64.5%	54.0%	
		Female	35	22	57	
			56.5%	35.5%	46.0%	
7.	Weight of the child*	N	62	62	124	.843
8.	Term/Preterm	Term	60	61	121	.559
			96.8%	98.4%	97.6%	
		Preterm	2	1	3	
			3.2%	1.6%	2.4%	

Out of the 62 mothers in the intervention group, 3 of them were between the ages of 15-20 years (4.8%), 27 were between 21-25 years of age (43.5%), 24 were between 26-30 years of age (38.7%) and 8 were between 31-35 years of age (12.9%). 59 participants had number of family members ranging between 1-5 (95.2%) and 3 participants had between 6-10 (4.8%). Majority of the study participants in the intervention group had studied up to higher secondary (41.9%) and had a family income of Rs.10000-15000 (54.8%). Majority of them had a cesarean section (66.1%) and delivered a term baby (98.4%). Majority of the newborns in the intervention group were males (64.5%). Out of the 62 newborns in the intervention group, 2 had a birth weight ranging between 1-1.9 kg (3.2%), 39 between 2-2.9 kg (62.9%) and 21 between 3-3.9 kg (33.9%).

### Analysis of mother’s knowledge about prevention of neonatal hypothermia

Table 2 shows what mothers know about preventing newborn hypothermia. Of the participants in the control group, 75.8% were unaware of how to maintain the body heat of the newborn, and 91.9% were unsure of which part of the body the baby is most prone to heat loss. This demonstrates that the majority of mothers were ignorant of the need to keep their babies’ bodies warm. 90.3% of participants were unaware of which body parts become cold when a newborn looses heat and 96.8% of participants were unsure of which body part to cover to preserve the infant’s body heat. This data shows that majority of the mothers were not aware of thermal care of the newborn and thus did not know how to prevent neonatal hypothermia. The majority of participants (83.9%) were aware of the best time for the baby’s first bath. Just 21.0% and 29.0%, respectively, knew the best way to dry the newborn after delivery and the ideal time of day to bathe the baby. It may be inferred from this that while a lot of mothers knew when it was best to give their baby their first bath, they were less aware of the best time of day to do so and how to properly dry their infant. Among the participants, a considerable percentage (64.5%) used benzoin resin vapours to dry the baby, indicating that many mothers were unaware that this practice could lead to upper respiratory tract infections in the infant. 75.8% were unaware of how frequently a baby should have their dirty nappy or cloth changed. As wearing a wet, soiled nappy for an extended period of time is one of the causes of hypothermia, unaware mothers put their newborns at risk for the condition. The majority did not know how often the baby should be breastfed (59.7%) or when the newborn needed to start breastfeeding (75.8%). This indicates that most also didn’t know enough about breastfeeding, which is a vital part of preventing hypothermia. Merely 40.3% of them were aware of how frequently a normal baby’s weight should be monitored following delivery. 96.8% of them were aware that the best way to keep body temperature stable just after birth was skin to skin contact. 95.2% of the participants knew which type of bath to follow. It suggests that intervention is not necessary in this case because almost all mothers are aware of the benefits of skin-to-skin contact and the style of bathing that is best for a baby.

**Table 2.** Mothers’ knowledge for preventing neonatal hypothermia.

Sl.no.	Knowledge domain	Response	Non-Intervention group	Intervention group	Total	P*
1.	Baby is prone to maximum heat loss from which part of the body	Accurate	5	62	67	.000
			8.1%	100.00%	54.0%	
		Inaccurate	57	0	57	
			91.9%	0.0%	46.0%	
2.	How can you maintain body heat of the baby	Accurate	15	44	59	.000
			24.2%	71.0%	47.6%	
		Inaccurate	47	18	65	
			75.8%	29.0%	52.4%	
3.	Which part of baby’s body should be covered mainly to conserve heat	Accurate	2	61	63	.000
			3.2%	98.4%	50.8%	
		Inaccurate	60	1	61	
			96.8%	1.6%	49.2%	
4.	Which of the following body parts will be cold when baby looses heat	Accurate	6	0	6	.012
			9.7%	0.0%	4.8%	
		Inaccurate	56	62	118	
			90.3%	100.0%	95.2%	
5.	Best method to maintain body heat immediately after birth	Accurate	60	62	122	.154
			96.8%	100.0%	98.4%	
		Inaccurate	2	0	2	
			3.2%	0.0%	1.6%	
6.	When ideally baby’s first bath should be given	Accurate	52	62	114	.001
			83.9%	100.0%	91.9%	
		Inaccurate	10	0	10	
			16.1%	0.0%	8.1%	
7.	What type of bath is preferred for a newborn	Accurate	59	62	121	.080
			95.2%	100.0%	97.6%	
		Inaccurate	3	0	3	
			4.8%	0.0%	2.4%	
8.	At what time of the day will you bathe the baby	Accurate	18	40	58	.000
			29.0%	64.5%	46.8%	
		Inaccurate	44	22	66	
			71.0%	35.5%	53.2%	
9.	How do you dry the baby after bath	Accurate	13	45	58	.000
			21.0%	72.6%	46.8%	
		Inaccurate	49	17	66	
			79.0%	27.4%	53.2%	
10.	Do you use benzoin resin fumes/sambrani/dhoop to dry the baby	Yes	40	3	43	.000
			64.5%	4.8%	34.7%	
		No	22	59	81	
			35.5%	95.2%	65.3%	
11.	How often do you change baby’s soiled clothes/diaper	Accurate	15	42	57	.000
			24.2%	67.7%	46.0%	
		Inaccurate	47	20	67	
			75.8%	32.3%	54.0%	
12.	When should breastfeeding be initiated in the newborn	Accurate	15	53	68	.000
			24.2%	85.5%	54.8%	
		Inaccurate	47	9	56	
			75.8%	14.5%	45.2%	
13.	How often do you breastfeed the baby	Accurate	25	48	73	.000
			40.3%	77.4%	58.9%	
		Inaccurate	37	14	51	
			59.7%	22.6%	41.1%	
14.	How often should weight of normal baby be checked after delivery	Accurate	25	50	75	.000
			40.3%	80.6%	60.5%	
		Inaccurate	37	12	49	
			59.7%	19.4%	39.5%	

Post intervention, 71.0% of the participants understood how to keep the baby’s body temperature stable, and all of them knew which part of the body the newborn is most likely to loose heat from (100%; p-value = 0.000). This demonstrates that mothers’ understanding of newborn thermal care has improved as a result of the video-based intervention. Of the participants, 98.4% (p-value = 0.000) recognised which body part to cover to retain the baby’s body heat, however none of them knew which body parts (0%; p-value = 0.012) get cold when the baby loses heat. Following the intervention, mothers were more informed about which parts of the infant to cover to retain heat, but they lacked sufficient knowledge about which parts of the body get cold in the event of hypothermia. Every participant was aware of the best timing for the baby’s first bath (100%; p-value = 0.001). The majority of them were aware of the best time of day to bathe the infant (64.5%; p-value = 0.000) and the best technique for drying the newborn (72.6%; p-value = 0.000). This proves that the intervention has aided numerous mothers in learning the proper technique to bathe their infants so as not to alter the baby’s body temperature and increase their risk of hypothermia. The majority of participants (95.2%; p-value = 0.000) did not use benzoin resin fumes to dry the baby. Of them, 67.7% (p-value = 0.000) were aware of the frequency of changing a baby’s soiled nappy or cloth. This indicates that mothers were also instructed on the usage of benzoin fumes and the importance of changing dirty diapers often as part of the video-based intervention. The majority of women knew when to start breastfeeding a newborn (85.5%; p-value = 0.000) and how often to do so (77.4%; p-value = 0.000), suggesting that the intervention had enhanced mothers’ breastfeeding knowledge. Of them, 80.6% (p-value = 0.000) were aware of the frequency of weight checks for a normal baby. The intervention has led to a significant number of mothers to know how often the baby’s weight should be checked.

Responses to questions on ideal time of the day for baby’s bath, changing soiled diapers and breastfeeding in Table 2 were similar to responses found by Leilane Barbosa de Sousa et al on their study on effect of educational video on newborn care knowledge among mothers. ^31^ Knowledge of mothers about newborn’s first bath, drying the baby and initiation of breastfeeding in Table 2 were similar to results found in a study by Richard Mangwi et al. ^32^

Responses to questions on best method to maintain body temperature immediately after birth and type of bath to be preferred was found to be statistically insignificant, showing that the mothers had adequate knowledge regarding this and do not require an intervention.

### Analysis of mother’s practices about prevention of neonatal hypothermia

Mothers’ preventive measures against neonatal hypothermia are shown in Table 3. Seventy-seven percent of the mothers in the control group did not wrap their baby with a shirt, cap, gloves, socks, nappies, or towel. The majority of them (95.2%) do not examine the baby’s legs and abdomen for heat loss and (64.5%) do not cover the baby’s head with a cap. This demonstrates that most mothers do not sufficiently cover their babies and do not routinely check for neonatal hypothermia danger signs. 83.9% of them lie down in bed with their baby next to them, suggesting that a large number of the mothers engaged in skin-to-skin contact. 90.3% of women do not clean their breasts before breastfeeding. This indicates that the majority of mothers do not nurse with proper hygiene, leaving baby susceptible to diseases. Only breastfeeding is provided for the infant by 71.0% of mothers, and in 98.4% of these cases, the majority of the areola and nipple are in the baby’s mouth during feeding. A sizable percentage of mothers ensure appropriate attachment throughout breastfeeding and provide exclusive breastfeeding. 100% of the babies slept well after feeding. This demonstrates that every mother gave her baby an appropriate breastfeed.

**Table 3.** Mothers’ practices for preventing neonatal hypothermia.

Sl.no.	Practice	Response	Non-Intervention Group	Intervention Group	Total	P*
1.	Mother dresses the baby with shirt, cap, gloves, socks, nappy and wrap with towel.	Yes	14	33	47	.000
			22.6%	53.2%	37.9%	
		No	48	29	77	
			77.4%	46.8%	62.1%	
2.	Mother covers baby’s head with cap.	Yes	22	61	83	.000
			35.5%	98.4%	66.9%	
		No	40	1	41	
			64.5%	1.6%	33.1%	
3.	Mother and baby lie down together in a bed.	Yes	52	62	114	.001
			83.9%	100.0%	91.9%	
		No	10	0	10	
			16.1%	0.0%	8.1%	
4.	Mother checks baby’s abdomen and legs for heat loss.	Yes	3	46	49	.000
			4.8%	74.2%	39.5%	
		No	59	16	75	
			95.2%	25.8%	60.5%	
5.	Mother cleans the breast before breastfeeding.	Yes	6	34	40	.000
			9.7%	54.8%	32.3%	
		No	56	28	84	
			90.3%	45.2%	67.7%	
6.	Mother washes hands before breastfeeding.	Yes	0	11	11	0.001
			0.0%	17.7%	8.9%	
		No	62	51	113	
			100.0%	82.3%	91.1%	
7.	Mother provides only breastfeeding for the baby.	Yes	44	52	96	.086
			71.0%	83.9%	77.4%	
		No	18	10	28	
			29.0%	16.1%	22.6%	
8.	Most of areola and nipple is in baby’s mouth while feeding the baby.	Yes	61	62	123	.315
			98.4%	100.0%	99.2%	
		No	1	0	1	
			1.6%	0.0%	0.8%	
9.	Baby sleeps well after feeding.	Yes	62	62	124	-
			0.0%	0.0%	0.0%	
		No	-	-	-	

Following the intervention, 53.2% of the mothers dressed their baby in a shirt, cap, gloves, socks, diaper and towel (p-value = 0.000). The majority of them checked the baby’s legs and abdomen for heat loss (74.2%; p-value = 0.000) and covered the baby’s head with a cap (98.4%; p-value = 0.000). Making mothers adequately cover their babies has been a successful outcome of the intervention. Additionally, it has effectively prompted most mothers to look for warning signs in their newborns. This will encourage prompt medical attention, preventing the potentially fatal effects of neonatal hypothermia. Every mother lies down in bed with her baby (100%; p-value = 0.001), indicating that the intervention has improved mothers’ practices regarding skin-to-skin contact. Before breastfeeding, the majority of women clean their breasts (54.8%; p-value = 0.000). 100% of the babies slept soundly after feeding. Enhancing hygienic breastfeeding practices and ensuring adequate breastfeeding have been made possible by the video-based intervention.

Practices regarding adequately covering the baby, identifying danger signs of neonatal hypothermia and adequate breastfeeding were similar to results found by Swathi Eluri et al. ^33^ Practices of mothers on skin to skin contact, exclusive breastfeeding and hygienic breastfeeding practices in Table 3 were comparable to results found by Laura Subramanian et al. ^34^

Intervention group had better exclusive breastfeeding practices as compared to control group, but not statistically significant. This data indicates that the mothers were practicing exclusive breastfeeding and an intervention is not required for that. This finding was similar to results found by B. Adhisivam et al in a study assessing the impact of a video based health education program. ^35^

## Critical reflection

Of the 124 moms who took part in our study, 97.6% of them gave birth to a term child. The intervention increased mothers’ knowledge from 24.2% to 71.0% regarding how to maintain their baby’s body heat. Mothers in the intervention group knew when to start breastfeeding at a rate of 85.5%, compared to just 24.2% of mothers in the control group.

Following the intervention, mothers’ practices for controlling the newborn’s body temperature, maintaining skin-to-skin contact, and exclusively breastfeeding showed considerable improvement. In the control group, 75.8% of mothers were unaware of the appropriate time to start breastfeeding. 85.5% of mothers knew when to start breastfeeding their newborn after the intervention.

In the post-intervention group, 98.4% of mothers wore a cap over their baby’s head, compared to just 35.5% in the control group. Mothers’ knowledge and practices towards preventing neonatal hypothermia have improved as a result of the intervention, as seen by improvements in exclusive breastfeeding and newborn thermal care.

## Discussion

This study found that the video based intervention has been effective in improving the knowledge of mothers about breastfeeding, ideal time of the day for baby’s bath and changing of soiled diapers. This finding was consistent with the study done by Leilane Barbosa de Sousa et al in Brazil. ^31^ The intervention has helped mothers learn about initiation of breastfeeding, drying of the newborn and the baby’s first bath. These results aligned with those found by Richard Mangwi et al in Uganda. ^32^

We found that the video has been successful in improving good neonatal practices such as exclusive breastfeeding, thermal care of newborn, hand hygiene and recognising danger signs in newborn. These finding were comparable to the study conducted in Mangalore by Swathi Eluri et al. ^33^ Post intervention, skin to skin contact, exclusive breastfeeding and hygienic breastfeeding practices among mothers had improved, which was similar to results found by Laura Subramanian et al in India. ^34^

Better exclusive breastfeeding practices were observed in the intervention group as compared to the control group, but it was not statistically significant. This result indicates that most mothers were practicing exclusive breastfeeding and an intervention is not required for that. This was in line with results found by B. Adhisivam et al in South India. ^35^

### Limitations

The study was conducted only among primipara mothers with normal newborns and studies involving educating multipara mothers, mothers with severely ill newborns and mothers of newborns admitted in NICU may by required. This study was conducted in a public institution in an urban area. Hence further studies in public and private institutions of rural and urban areas are recommended.

## Conclusion

The results of this study demonstrate the significant improvement in mothers’ knowledge and practices about the prevention of neonatal hypothermia due to the use of a video-based educational tool. Consequently, it will result in a decrease in mortality and morbidity rates of neonates related to hypothermia.

## Ethics and consent

The Institutional Ethics Committee at Kasturba Medical College in Mangalore has granted ethical clearance on 16/03/23 (Protocol No: IECKMCMLR-03/2023/94). We have received approval from the Government Lady Goschen Hospital’s Medical Superintendent to carry out this research.

Informed written consent for participation in the study was obtained.

## Data Availability

Figshare: (Hypothermia FINAL DATA Excel), https://doi.org/10.6084/m9.figshare.26378965.v1. ^36^ Figshare: Questionnaire and Consent form.docx, https://doi.org/10.6084/m9.figshare.26402773.v1. ^37^ Data are available under the terms of the Creative Commons Attribution 4.0 International license (CC-BY 4.0).
