# Assessment of early neonatal practices to prevent hypothermia ; A comparative study

**DOI:** 10.12688/f1000research.154628.3

**Published:** 2024-11-06

**Authors:** Smrithi GM, Gayathri Renganathan, Rohith Motappa, Nitin Joseph, Ravikiran SR

**Affiliations:** 1 Kasturba Medical College Mangalore, Manipal Academy of Higher Education, Karnataka, Manipal, 576 104, India; 2 Senior Resident, Department of Paediatrics, Kasturba Medical College, Mangalore, Manipal Academy of Higher Education, Karnataka, Manipal, 576 104, India; 3 Assistant Professor, Department of Community Medicine, ESIC Medical College & PGIMSR and Model Hospital, Rajajinagar Bangalore-560010, Karnataka, India; 4 Associate Professor, Department of Community Medicine, Kasturba Medical College Mangalore, Manipal Academy of Higher Education, Karnataka, Manipal, 576 104, India; 5 Professor, Department of Paediatrics, Kasturba Medical College Mangalore, Manipal Academy of Higher Education, Karnataka, Manipal, 576 104, India

**Keywords:** Neonatal Hypothermia, Prevention, Video based education, Knowledge, Practice

## Abstract

**Background:**

Globally, neonatal deaths are significantly attributed to hypothermia. This is mostly because of its co-morbidity with asphyxia, premature birth and severe neonatal infections. Worldwide, neonatal hypothermia case fatality rates (CFRs) range from 8.5% to 52%. This study aimed to assess how well a video-based training intervention on mothers’ knowledge and practices in preventing neonatal hypothermia worked.

**Methods:**

The knowledge and practices of two groups of primi-para mothers—one control group and one intervention group—were compared in order to achieve this. A total of 124 primipara mothers took part in the research. Using a video based tool, the intervention group was educated about preventing hypothermia in newborns. Both control and intervention group mothers were interviewed to compare the knowledge and practices.

**Results:**

Sixty-one of the sixty-two mothers in the control group were unsure of which body area to cover in order to preserve the baby’s body heat. Following the intervention, 61 out of 62 mothers in the group recognised which body part to cover to protect the baby’s body heat. In the post-intervention group, 98.4% of moms wore a cap over their baby’s head, compared to just 35.5% in the control group.

**Conclusion:**

The results of this study demonstrate the significant improvement in mothers’ knowledge and actions about the prevention of neonatal hypothermia resulting from the use of a video-based training tool.

## Introduction

A core body temperature of less than 36.5 °C or a skin temperature of less than 36 °C is considered neonatal hypothermia, according to the World Health Organisation (WHO). ^1^ A baby is said to have moderate hypothermia if their temperature is between 32.0 and 34.9°C (89.6 and 96.6°F), and severe hypothermia if it is lower. ^2^ Hypothermia is a significant contributor to newborn fatalities globally, even though it seldom ends in death. This is mainly due to its co-morbidity with severe neonatal infections, premature birth, and asphyxia. ^3^ Hypothermia engenders a vicious cycle with physiological abnormalities such as hypoxia, hypoglycemia and shock. ^4^

Every year, an estimated four million newborns die. ^5^ Over 99 percent of newborn fatalities take place in underdeveloped nations. One million babies perish in the first month of birth each year, with India alone bearing one-fourth of the burden. ^6^ The case fatality rates (CFR) for newborn hypothermia vary from 8.5% to 52% globally. ^3^ According to a study by Wake et al, neonates with hypothermia had nearly five times the risk of dying as newborns than those with a normal body temperature. ^6^ This study will help achieve the Sustainable Development Goal of the UN, which is to guarantee healthy lives and promote well-being for everyone at all ages. ^7^

Teaching moms how to recognise the symptoms of newborn hypothermia and how to prevent it can be a crucial preventive measure. Only during their hospital stay do newborns have medical supervision. Therefore, it is essential to educate mothers who constantly keep a watch on and tend to the newborn. Researching the effects of maternal education will help reduce newborn hypothermia in general.

## Summary of evidence

Anurag Srivastava et al. carried out a cross-sectional study among new mothers in Western Uttar Pradesh to ascertain the knowledge and practice gaps on newborn thermal care. It was shown that 60.9% of those surveyed knew exactly how to keep a newborn warm after delivery. ^8^

Mahvish Qazi et al. did a study in Northern India on mothers’ awareness of how to prevent neonatal hypothermia. It was shown that while 45% of moms knew how to rapidly wipe a newborn to keep them warm, just 3% of mothers were familiar with kangaroo mother care. ^9^

In Jimma, South West Ethiopia, a research by Kebede et al. indicated that 32.5% of moms practiced early newborn bathing. Since it causes newborn hypothermia and related problems, this needs to be addressed first. ^10^

Nearly half of mothers lack sufficient knowledge about preventing hypothermia, according to a cross-sectional study on the topic conducted by Indu Shah et al. among 70 mothers of newborns. ^11^

In the SNCU of M.K.C.G Medical College Hospital, 54 mothers of low birth weight neonates participated in a study by Sadhana Panda et al. on their understanding of preventing newborn hypothermia. Just 44% of mothers believed that drying the infant right after would stop heat loss, and only 11% of mothers knew enough about skin-to-skin contact, the most effective way to stop heat loss. ^12^

According to a study by Ahmed et al. in three hospitals in the state of Khartoum, only 52.66% of mothers were in favour of treating newborn hypothermia symptoms as soon as they appeared. ^13^

A research by Kibaru et al. in the Nakuru Central district of Kenya found that just 9.7% of mothers recognised hypothermia as a potential danger sign for their newborn. ^14^ According to a research conducted in rural Bangladesh by Zaman et al., hypothermia was the infant risk indication that newly delivered women (RDW) identified the least often(26.1%). ^15^

Smita Srivastava et al.’s randomised control trial found that skin-to-skin contact during the initial postpartum period is associated with improved neonatal temperature regulation. ^16^ Focused interventions reduced the average monthly risk of hypothermia among low birth weight and late preterm neonates to 13.3%, according to research by Christine Andrews et al. Prior to this, the average monthly rate of hypothermia was 29.8%. ^17^

Infants who did not receive their mothers’ first skin-to-skin contact during delivery had a 4.3-fold increased risk of developing hypothermia, as per research by Demissie et al. ^18^

To determine how well kangaroo mother care (KMC) works to keep low birth weight babies warm while being transported from the hospital to their families, Nimbalkar et al. undertook a study. After being discharged from the hospital, the study group experienced a mean rise in temperature of 0.01°C, whereas the control group saw an average decrease in temperature of 0.07°C during transportation. ^19^

As part of the intervention plan for their study, Noel Joseph et al. added a number of interventions, such as key cards, hypothermia algorithms, and nursing education. Following implementation, the mother-baby unit’s incidence of hypothermia dramatically decreased, falling from 20.9% to 14.5%. Furthermore, there were 71% fewer newborns who required admission to the Neonatal Intensive Care Unit (NICU). ^20^

Mukunya et al.’s multivariable analysis revealed that delayed breastfeeding initiation, low birth weight and home birth were the variables linked to a higher risk of newborn hypothermia. ^21^

According to a study by Olawuyi et al., nearly two thirds (66.7%) of mothers in newborn intensive care units were aware of the advantages of Kangaroo Mother Care (KMC). ^22^

In a research by S P Choudhary et al., which involved medical and paramedical personnel, only 47.8% of the participants correctly defined newborn hypothermia, and a noteworthy 52.2% thought it was an uncommon problem. ^23^

In the early postpartum period, when neonates are most susceptible to hypothermia, the warm chain was not continuously maintained, according to a study by Karsten Lunze et al done in rural Zambia. ^24^

Within the first five minutes of birth, 27% of newborns were wrapped and 42% of neonates were dried, according to a study conducted in rural southern Tanzania by Donat Shamba et al. The fact that carers neglected to tend to the baby until after the placenta was delivered was the main cause of the delay in wrapping and drying. ^25^

Early newborn washing was practiced by 87.5% of women who did not visit health facilities for ANC, compared to 54.8% of moms who had four or more ANC visits, according to Gebrew Getachew et al. ^26^

The results of a study by Luke Mullany et al showed that infants whose breastfeeding was delayed for longer than 24 hours were almost 50% more likely to experience hypothermia. ^27^ According to a research by Meseret Ekubay et al, 58.3% of mothers started breastfeeding their newborns in the first hour following birth. ^28^

According to Golicha Wako et al., out of 388 mothers in South Ethiopia, 69.1% of them covered their newborns’ heads right away after delivery, and almost all of them did so for duration of the neonatal period. ^29^

Dongre et al undertook a study to determine how well-informed mothers in periurban Wardha were about infant warning signs. Just 2.8% of respondents identified hypothermia in neonates as one of the warning indicators. ^30^

In health education, video based learning provides an effective teaching medium for knowledge acquisition, skill development, and attitude modification, according to a systematic review and meta analysis by Mariano Margado et al. Video based learning leads to substantial improvement in medical awareness. ^31^

## Need for study

No study has been conducted which shows the efficacy of a simple video based educational intervention on preventing neonatal hypothermia. In line with pertinent theories of attitude change and health behavior, video-based learning exhibits promise in the field of health education. ^31^ This study will show the effectiveness of a video based educational tool in educating mothers and will be useful for the primipara mothers to gain adequate knowledge about preventing neonatal hypothermia.

### Methods

The study was conducted in the postnatal wards of Government Lady Goschen Hospital, Mangalore. It was a non randomized educational trial. Primipara mothers of newborns admitted in the postnatal wards of Government Lady Goschen Hospital, Mangalore were identified and included in the study. They were divided into Group A (control) and Group B (case), who would receive counselling on preventing neonatal hypothermia via a video-based educational tool. The inclusion criteria for selection was primipara mothers of newborns in the postnatal ward who were willing to participate in the study. Exclusion criteria included mothers of severely ill newborns, mothers whose babies were admitted in NICU, mothers who had home deliveries, inability of mother to participate Eg: mother is severely ill/stressed at the time of study and mothers who are not willing to participate.

The study was conducted over a period of 2 months. 124 primipara mothers were identified and divided into two groups of 62 each. This sample size was derived using an article based on new born thermal care as reference. ^8^

$$n=\frac{{\left(\;Z\;\alpha \;\right)}^{2}\;\mathrm{pq}\;}{d^{2}}$$

A structured questionnaire about preventing hypothermia in neonates was used as tool for data collection. Following a review of literature and expert advice, the semi-structured questionnaire was developed. Validation of the content was completed.

Participants in Group A (control) did not get any intervention and were recruited between June 24, 2023 and July 24, 2023. Group B participants were educated on preventing hypothermia in newborns using video based intervention and were recruited between July 25, 2023 and August 25, 2023.

The educational video was recorded using images and with voiceover to educate mothers on preventing hypothermia in the neonatal period. The duration of the video was 6 minutes. It has been taken in the local language. The video was displayed on the projector screen in the demo room attached to the post natal ward separately. The display resolution was HDR. Each mother was shown the video separately (single blinded) to ensure one-on-one interaction with the investigator. It was shown to the mothers in the intervention group on day 2 post delivery. This was to ensure that the mother is not in any pain or stress and is mentally fit and comfortable to understand the contents of the video. Group B was interviewed twenty-four hours after they were educated about preventing neonatal hypothermia to improve the validity of understanding.

Participants’ questions on preventing newborn hypothermia were addressed. The data collection tool was the same questionnaire used for both the groups. The principal investigator conducted interviews with the mothers in order to collect data.

Data was entered in Microsoft Excel 2016 (Microsoft Excel, RRID:SCR_016137) and analyzed using SPSS version 25 (IBM SPSS Statistics, RRID:SCR_019096). Data was interpreted in proportions and percentages, and Chi-square and P-values were obtained.

## Results

Table 1 depicts the demographic information of study participants. In our study there were a total of 124 participants, out of which 50% belonged to the intervention group.

**Table 1.** Demographic characteristics.

Sl. number	Demographical characteristic	Response	Non-Intervention group	Intervention group	Total	P*
1.	Age of mother, years Mean (IQR)*	N	62	62	124	.607
2.	Maternal education	Illiterate	1	0	1	.159
			1.6%	0.0%	0.8%	
		1 ^st^ – 10 ^th^ std	29	23	52	
			46.8%	37.1%	41.9%	
		Higher secondary	15	26	41	
			24.2%	41.9%	33.1%	
		Graduate and above	17	13	30	
			27.4%	21.0%	24.2%	
3.	Type of delivery	Normal Vaginal	36	21	57	.007
			58.1%	33.9%	46.0%	
		Cesarean Section	26	41	67	
			41.9%	66.1%	54.0%	
4.	Family income	Less than 5000 rs	1	0	1	.012
			1.6%	0.0%	0.8%	
		5000-10000 rs	42	26	68	
			67.7%	41.9%	54.8%	
		10000-15000 rs	19	34	53	
			30.6%	54.8%	42.7%	
		More than 15000 rs	0	2	2	
			0.0%	3.2%	1.6%	
5.	Number of members in the family*	N	62	62	124	.094
6.	Sex of the child	Male	27	40	67	.019
			43.5%	64.5%	54.0%	
		Female	35	22	57	
			56.5%	35.5%	46.0%	
7.	Weight of the child*	N	62	62	124	.843
8.	Term/Preterm	Term	60	61	121	.559
			96.8%	98.4%	97.6%	
		Preterm	2	1	3	
			3.2%	1.6%	2.4%	

51 participants had number of family members ranging between 1-5 (82.3%) and rest had between 6-10 (17.7%). Majority of them had a normal vaginal delivery (58.1%) and delivered a term baby (96.8%). Out of the 62 mothers in the intervention group, 59 participants had number of family members ranging between 1-5 (95.2%) and 3 participants had between 6-10 (4.8%). Majority of the study participants in the intervention group had studied up to higher secondary (41.9%). Majority of them had a cesarean section (66.1%) and delivered a term baby (98.4%).

### Analysis of mother’s knowledge about prevention of neonatal hypothermia

Table 2 shows what mothers know about preventing newborn hypothermia. Of the participants in the control group, 75.8% were unaware of how to maintain the body heat of the newborn. 91.9% were unsure of which part of the body the baby is most prone to heat loss. 90.3% of participants were unaware of which body parts become cold when a newborn looses heat. Among the participants, a considerable percentage (64.5%) used benzoin resin vapours to dry the baby. The majority did not know when to start breastfeeding (75.8%). Post intervention, 71.0% of the participants understood how to keep the baby’s body temperature stable, and all of them knew which part of the body the newborn is most likely to loose heat from (100%; p-value = 0.000). The majority of women knew when to start breastfeeding a newborn (85.5%; p-value = 0.000).

**Table 2.** Mothers’ knowledge for preventing neonatal hypothermia.

Sl.no.	Knowledge domain	Response	Non-Intervention group	Intervention group	Total	P*
1.	Baby is prone to maximum heat loss from which part of the body	Accurate	5	62	67	.000
			8.1%	100.00%	54.0%	
		Inaccurate	57	0	57	
			91.9%	0.0%	46.0%	
2.	How can you maintain body heat of the baby	Accurate	15	44	59	.000
			24.2%	71.0%	47.6%	
		Inaccurate	47	18	65	
			75.8%	29.0%	52.4%	
3.	Which part of baby’s body should be covered mainly to conserve heat	Accurate	2	61	63	.000
			3.2%	98.4%	50.8%	
		Inaccurate	60	1	61	
			96.8%	1.6%	49.2%	
4.	Which of the following body parts will be cold when baby looses heat	Accurate	6	0	6	.012
			9.7%	0.0%	4.8%	
		Inaccurate	56	62	118	
			90.3%	100.0%	95.2%	
5.	Best method to maintain body heat immediately after birth	Accurate	60	62	122	.154
			96.8%	100.0%	98.4%	
		Inaccurate	2	0	2	
			3.2%	0.0%	1.6%	
6.	When ideally baby’s first bath should be given	Accurate	52	62	114	.001
			83.9%	100.0%	91.9%	
		Inaccurate	10	0	10	
			16.1%	0.0%	8.1%	
7.	What type of bath is preferred for a newborn	Accurate	59	62	121	.080
			95.2%	100.0%	97.6%	
		Inaccurate	3	0	3	
			4.8%	0.0%	2.4%	
8.	At what time of the day will you bathe the baby	Accurate	18	40	58	.000
			29.0%	64.5%	46.8%	
		Inaccurate	44	22	66	
			71.0%	35.5%	53.2%	
9.	How do you dry the baby after bath	Accurate	13	45	58	.000
			21.0%	72.6%	46.8%	
		Inaccurate	49	17	66	
			79.0%	27.4%	53.2%	
10.	Do you use benzoin resin fumes/sambrani/dhoop to dry the baby	Yes	40	3	43	.000
			64.5%	4.8%	34.7%	
		No	22	59	81	
			35.5%	95.2%	65.3%	
11.	How often do you change baby’s soiled clothes/diaper	Accurate	15	42	57	.000
			24.2%	67.7%	46.0%	
		Inaccurate	47	20	67	
			75.8%	32.3%	54.0%	
12.	When should breastfeeding be initiated in the newborn	Accurate	15	53	68	.000
			24.2%	85.5%	54.8%	
		Inaccurate	47	9	56	
			75.8%	14.5%	45.2%	
13.	How often do you breastfeed the baby	Accurate	25	48	73	.000
			40.3%	77.4%	58.9%	
		Inaccurate	37	14	51	
			59.7%	22.6%	41.1%	
14.	How often should weight of normal baby be checked after delivery	Accurate	25	50	75	.000
			40.3%	80.6%	60.5%	
		Inaccurate	37	12	49	
			59.7%	19.4%	39.5%	

### Analysis of mother’s practices about prevention of neonatal hypothermia

Mothers’ preventive measures against neonatal hypothermia are shown in Table 3. 77% of the mothers in the control group did not wrap their baby with a shirt, cap, gloves, socks, nappies, or towel. The majority of them (95.2%) do not examine the baby’s legs and abdomen for heat loss and (64.5%) do not cover the baby’s head with a cap. Following the intervention, 53.2% of the mothers dressed their baby in a shirt, cap, gloves, socks, diaper and towel (p-value = 0.000). The majority of them checked the baby’s legs and abdomen for heat loss (74.2%; p-value = 0.000) and covered the baby’s head with a cap (98.4%; p-value = 0.000).

**Table 3.** Mothers’ practices for preventing neonatal hypothermia.

Sl.no.	Practice	Response	Non-Intervention Group	Intervention Group	Total	P*
1.	Mother dresses the baby with shirt, cap, gloves, socks, nappy and wrap with towel.	Yes	14	33	47	.000
			22.6%	53.2%	37.9%	
		No	48	29	77	
			77.4%	46.8%	62.1%	
2.	Mother covers baby’s head with cap.	Yes	22	61	83	.000
			35.5%	98.4%	66.9%	
		No	40	1	41	
			64.5%	1.6%	33.1%	
3.	Mother and baby lie down together in a bed.	Yes	52	62	114	.001
			83.9%	100.0%	91.9%	
		No	10	0	10	
			16.1%	0.0%	8.1%	
4.	Mother checks baby’s abdomen and legs for heat loss.	Yes	3	46	49	.000
			4.8%	74.2%	39.5%	
		No	59	16	75	
			95.2%	25.8%	60.5%	
5.	Mother cleans the breast before breastfeeding.	Yes	6	34	40	.000
			9.7%	54.8%	32.3%	
		No	56	28	84	
			90.3%	45.2%	67.7%	
6.	Mother washes hands before breastfeeding.	Yes	0	11	11	0.001
			0.0%	17.7%	8.9%	
		No	62	51	113	
			100.0%	82.3%	91.1%	
7.	Mother provides only breastfeeding for the baby.	Yes	44	52	96	.086
			71.0%	83.9%	77.4%	
		No	18	10	28	
			29.0%	16.1%	22.6%	
8.	Most of areola and nipple is in baby’s mouth while feeding the baby.	Yes	61	62	123	.315
			98.4%	100.0%	99.2%	
		No	1	0	1	
			1.6%	0.0%	0.8%	
9.	Baby sleeps well after feeding.	Yes	62	62	124	-
			0.0%	0.0%	0.0%	
		No	-	-	-	

## Critical reflection

Of the 124 moms who took part in our study, 97.6% of them gave birth to a term child. The intervention increased mothers’ knowledge from 24.2% to 71.0% regarding how to maintain their baby’s body heat. Mothers in the intervention group knew when to start breastfeeding at a rate of 85.5%, compared to just 24.2% of mothers in the control group.

Following the intervention, mothers’ practices for controlling the newborn’s body temperature, maintaining skin-to-skin contact, and exclusively breastfeeding showed considerable improvement. In the control group, 75.8% of mothers were unaware of the appropriate time to start breastfeeding. 85.5% of mothers knew when to start breastfeeding their newborn after the intervention.

In the post-intervention group, 98.4% of mothers wore a cap over their baby’s head, compared to just 35.5% in the control group. Mothers’ knowledge and practices towards preventing neonatal hypothermia have improved as a result of the intervention, as seen by improvements in exclusive breastfeeding and newborn thermal care.

## Discussion

The demographic information in our study is depicted in Table 1. In the control group, 5 of them were between the ages of 15-20 years (0.08%), 29 were between 21-25 years of age (46.7%), 19 were between 26-30 years of age (30.6%) and 9 were between 31-35 years of age (14.5%). 51 participants belonged to family size of 1-5 members (82.3%) and 11 participants belonged to a family size of 6-10 (17.7%). Majority of them had studied between 1 ^st^ – 10 ^th^ std (46.8%) and had a family income of Rs.5000-10000 (67.7%) in the control group. Majority of them had a normal vaginal delivery (58.1%) and delivered a term baby (96.8%). Majority of the newborns in the control group were females (56.5%). Out of the 62 newborns in the control group, 6 had a birth weight ranging between 1-1.9 kg (9.7%), 30 between 2-2.9 kg (48.4%) and 26 between 3-3.9 kg (41.9%).

In the intervention group, 3 of them were between the ages of 15-20 years (4.8%), 27 were between 21-25 years of age (43.5%), 24 were between 26-30 years of age (38.7%) and 8 were between 31-35 years of age (12.9%). 59 participants had number of family members ranging between 1-5 (95.2%) and 3 participants had between 6-10 (4.8%). Majority of the study participants in the intervention group had studied up to higher secondary (41.9%) and had a family income of Rs.10000-15000 (54.8%). Majority of them had a cesarean section (66.1%) and delivered a term baby (98.4%). Majority of the newborns in the intervention group were males (64.5%). Out of the 62 newborns in the intervention group, 2 had a birth weight ranging between 1-1.9 kg (3.2%), 39 between 2-2.9 kg (62.9%) and 21 between 3-3.9 kg (33.9%).

Table 2 shows the analysis of mothers’ knowledge about preventing newborn hypothermia. Among the control group, a significant 96.8% of the mothers did not know which part of the baby's body should be covered to conserve heat. 91.9% of them did not know which part of the body is most susceptible to heat loss. Additionally, 90.3% could not identify the body part that will become cold when the baby loses heat. This indicates that majority of the mothers lack knowledge regarding three main contributing factors of hypothermia: the importance of covering the baby to conserve heat, identifying the areas of maximum heat loss, and recognizing which body parts need to be checked for hypothermia. This shows that the predominant cause of neonatal hypothermia can be prevented if the mothers had knowledge on how it occurs, how to check for it, and how to prevent it. Furthermore, 79.0% of them also did not know the proper method to dry the baby after bath. It suggests that they were unaware that bathing significantly reduce the body temperature and if the baby is not adequately dried, it can predispose to hypothermia. Moreover, 75.8% did not know how to maintain the body heat of the baby, and similarly, 75.8% of them did not know how often to change the baby's soiled clothes or diaper. This is brings to light, another easily preventable cause of neonatal hypothermia. If the mothers were made aware, that soiled clothes can cause hypothermia, they will change the soiled clothes immediately which can significantly reduce hypothermia.

Additionally, 75.8% of them did not know when breast feeding should be initiated in the newborns. 71% of the mothers were unaware of the ideal time of the day to bathe the baby. Notably, 64% of the mothers used benzoin resin fumes to dry the baby. These questions help address the fundamental aspects in preventing hypothermia. Highlighting and educating mothers about the timely initiation of breastfeeding, right time to bathe the baby, and risks of using benzoin resin to dry the baby, can help to formulate effective practices aimed at preventing hypothermia. Moreover, 59.7% of the mothers also did not know how often to breastfeed the baby, and a similar percentage of 59.7% did not know how often the weight of the baby must be checked. This highlights that the lack of maternal knowledge was the primary factor for the occurrence of neonatal hypothermia, which can otherwise be easily detected.

However, 96.8% of them knew the best practices to maintain body heat immediately after birth and 95.2% understood preferred bathing method for the newborn. This information suggests that the majority of the mothers understood the significance of maintaining the body temperature immediately after birth. Furthermore, 83.9% of the mothers also knew the ideal time for baby’s first bath. This indicates that most of the mothers had knowledge about the effects of bathing on body temperature and how hypothermia can be prevented by knowing the ideal time for the first bath of the baby.

In the intervention group we can see a significant improvement in the knowledge where 100% of the mothers now understood which part of the baby’s body is prone to maximum heat loss (p-value = 0.000), which body parts become cold when the baby loses heat (p-value= 0.12), the ideal time for first bath of the baby (p-value = 0.001), the type of bath that is preferred for the newborn (p-value = 0.080), and the most effective methods to maintain body heat immediately after birth (p-value = 0.154). Additionally,98.4% of the mothers knew the right part of the baby's body that must be covered mainly to conserve heat (p-value = 0.000). Furthermore, 95.2% of the mothers knew that benzoin resin fumes should not be used to dry the baby (p-value = 0.000) after the education session. A significant 85.5% knew the right time to initiate breast feeding in the newborn (p-value = 0.000). Again, 80.6% knew how frequently the weight of the baby should be checked after delivery (p-value = 0.000) and 77.4% knew the correct frequency of breastfeeding (p-value = 0.000). In this group, 72.6% of the mothers were aware of the right method to dry the baby after bathing (p-value = 0.000). after the intervention, 71.0% of the mothers also knew how to maintain the body heat of the baby (p-value = 0.000). Notably, 67.7% of the mothers knew how often a baby's soiled clothes or diapers needed to be changed (p-value = 0.000). Lastly, 64.5% of the mothers in this group knew the right time of the day to bathe the baby (p-value = 0.000). Responses to questions on ideal time of the day for baby’s bath, changing soiled diapers and breastfeeding in Table 2 were similar to responses found by Leilane Barbosa de Sousa et al on their study on effect of educational video on newborn care knowledge among mothers. ^32^ Knowledge of mothers about newborn’s first bath, drying the baby and initiation of breastfeeding in Table 2 were similar to results found in a study by Richard Mangwi et al. ^33^

Table 3 shows the assessment of mothers’ newborn practices. In the control group, all 100% of the mothers were noted to not wash hands prior to breastfeeding which is a significant finding. This highlights the importance of educating the mothers regarding possible infections and sepsis of the baby which can lead to hypothermia. Notably, 95.2% of mothers do not check the baby's abdomen and legs for heat loss. This implies that there is a significant lack of knowledge on how to check the baby for hypothermia. A majority of 90.3% of the mothers also failed to clean the breast before breastfeeding the baby. This again highlights the lack of knowledge regarding the possibility of the baby acquiring an infection. Furthermore, 77.4% of the mothers failed to dress the baby with a shirt, cap, gloves, socks, nappy, and wrap with towel. A considerable 64.5% of mothers did not cover the baby’s head with a cap. This underscores the importance of educating the mothers about appropriate clothing and covering to prevent heat loss in the babies. However, in 98.4% of the mothers, most of the areola and nipple were positioned effectively while feeding the baby. This implies that mothers knew the correct positioning of the baby for breastfeeding.

In the intervention group. 100% of the mothers knew that most of the areola and nipple must be inside the baby’s mouth while feeding (p-value = 0.315), and 100% of the mother and baby shared the same bed (p-value = 0.001). This demonstrates the key changes in the post intervention group were right positioning of the baby while breastfeeding and rooming in was achieved in all the mothers. Notably, 98.4% of them knew about covering the baby’s head with a cap after the intervention (p-value = 0.000). Additionally, 83.9% of them provide exclusive breastfeeding to the baby (p-value = 0.086). This has helped in implementing a very important practice of exclusive breast feeding. Furthermore, in this group, 74.2 % of the mothers now knew to check the baby’s abdomen and legs for heat loss (p-value = 0.000). This suggests that the video based educational session has had a significant impact in educating the mothers on early detection of hypothermia. These findings underscore the efficacy of the video based intervention which has improved the mothers understanding of the causes, identification, and preventive practices of neonatal hypothermia. However, only 54.8% of the mothers cleaned the breast before breastfeeding even after the educational intervention (p-value = 0.000). This underlines the importance of repeated counselling to reemphasize the importance of hygienic practices while breast feeding. In this group, 53.2% of the mothers now knew to dress the baby with a shirt, cap, gloves, socks, nappy, and wrap with towel (p-value = 0.000). Moreover, 82.3% of the mothers still did not wash their hands before feeding the baby even after the educational intervention (p-value = 0.001). Nevertheless,100% of the babies slept soundly after feeding in both groups, indicating that the babies were receiving adequate breastfeeding which ensured good sleep.

Practices regarding adequately covering the baby, identifying danger signs of neonatal hypothermia and adequate breastfeeding were similar to results found by Swathi Eluri et al. ^34^ Practices of mothers on skin to skin contact, exclusive breastfeeding and hygienic breastfeeding practices in Table 3 were comparable to results found by Laura Subramanian et al. ^35^ Intervention group had better exclusive breastfeeding practices as compared to control group, but not statistically significant. This data indicates that the mothers were practicing exclusive breastfeeding and an intervention is not required for that. This finding was similar to results found by B. Adhisivam et al in a study assessing the impact of a video based health education program. ^36^

### Limitations

The study was conducted only among primipara mothers with normal newborns and studies involving educating multipara mothers, mothers with severely ill newborns and mothers of newborns admitted in NICU may by required. This study was conducted in a public institution in an urban area. Hence further studies in public and private institutions of rural and urban areas are recommended.

## Conclusion

The results of this study demonstrate the significant improvement in mothers’ knowledge and practices about the prevention of neonatal hypothermia due to the use of a video-based educational tool. Consequently, it will result in a decrease in mortality and morbidity rates of neonates related to hypothermia.

## Ethics and consent

The Institutional Ethics Committee at Kasturba Medical College in Mangalore has granted ethical clearance on 16/03/23 (Protocol No: IECKMCMLR-03/2023/94). We have received approval from the Government Lady Goschen Hospital’s Medical Superintendent to carry out this research.

Informed written consent for participation in the study was obtained.

## Data Availability

Figshare: (Hypothermia FINAL DATA Excel), https://doi.org/10.6084/m9.figshare.26378965.v1. ^37^ Figshare: Questionnaire and Consent form.docx, https://doi.org/10.6084/m9.figshare.26402773.v1. ^38^ Figshare: Neonatal hypothermia video, https://doi.org/10.6084/m9.figshare.27324081.v1. ^39^ Data are available under the terms of the Creative Commons Attribution 4.0 International license (CC-BY 4.0).
