# Supplementary material for: Assessment of early neonatal practices to prevent hypothermia ; A comparative study
Source: F1000Res. 2024 Nov 6;13:934. Originally published 2024 Aug 20. [Version 3] doi: 10.12688/f1000research.154628.3 (PMC11562740; doi:10.12688/f1000research.154628.3)
Supplement: Peer review history

# Reviewer report: Reviewer response for version 3

**DOI:** 10.5256/f1000research.174223.r338314

**Published:** 2024-11-09

**Authors:** Linus Olson

**Affiliations:** 1 Karolinska Institutet, Solna, Sweden; 2 Department of Women's and Children's Health, Karolinska Institutet, Stockholm, Sweden

Dear editor and Authors 

 I feel the manuscript is now ready for indexing

Is the work clearly and accurately presented and does it cite the current literature?

Partly

If applicable, is the statistical analysis and its interpretation appropriate?

Partly

Are all the source data underlying the results available to ensure full reproducibility?

Yes

Is the study design appropriate and is the work technically sound?

Yes

Are the conclusions drawn adequately supported by the results?

Partly

Are sufficient details of methods and analysis provided to allow replication by others?

Yes

Reviewer Expertise:

Hyper- and hypothermia for neonates, Infections public health in LMIC countries, treatment with HT for HIE

I confirm that I have read this submission and believe that I have an appropriate level of expertise to confirm that it is of an acceptable scientific standard.

# Reviewer report: Reviewer response for version 2

**DOI:** 10.5256/f1000research.173140.r332748

**Published:** 2024-10-26

**Authors:** Linus Olson

**Affiliations:** 1 Karolinska Institutet, Solna, Sweden; 2 Department of Women's and Children's Health, Karolinska Institutet, Stockholm, Sweden

Dear Authors 

 I highly appreciate your many good changes to this important manuscript. It looks much more scientific now and discussions is much more in the right section and result as well, but for this manuscript to have the right impact and recognition there are still some improvements to be done. 

 You say you have filmed slides and used text, English subtitle, and speech in local.. But what camera is used what programs used for the above slides and texts etc. Also please attach either the video or slides as a supplementary file. 

 Since the questionnaire used is good. I still want your to be more to the point, What affects the hypothermia first then all numbers and text about the control group and intervention, as other reviewer tried to say in last review. 

 I feel you need to have a "closed loop" ref.in your summer of evidence section since this is a crucial part in communication especially when using videos as communication tools. (you need to mention this in the manuscript). This is also important for why the need for study exist and your manuscript can have a great impact, both locally but also for the scientific community but mostly to reduce the risk for hypothermia or even save the children in risk globally.

Is the work clearly and accurately presented and does it cite the current literature?

Partly

If applicable, is the statistical analysis and its interpretation appropriate?

Partly

Are all the source data underlying the results available to ensure full reproducibility?

Yes

Is the study design appropriate and is the work technically sound?

Yes

Are the conclusions drawn adequately supported by the results?

Partly

Are sufficient details of methods and analysis provided to allow replication by others?

Yes

Reviewer Expertise:

Hyper- and hypothermia for neonates, Infections public health in LMIC countries, treatment with HT for HIE

I confirm that I have read this submission and believe that I have an appropriate level of expertise to confirm that it is of an acceptable scientific standard, however I have significant reservations, as outlined above.

# Response

**Published:** 2024-10-29

**Authors:** Smrithi GM

Author Response

 Smrithi GM

 Kasturba Medical College Mangalore,

 Manipal Academy of Higher Education,

 Karnataka,

 Manipal, 576 104,

 India

 Dear reviewer,

 Thank you for your valuable comments on our article. We have made the following changes.

 1. Reviewer’s comment : “You say you have filmed slides and used text, English subtitle, and speech in local.. But what camera is used what programs used for the above slides and texts etc. Also please attach either the video or slides as a supplementary file”.

 Response : We have included the video as a supplementary file under extended data section.

 2. Reviewer’s comment : “Since the questionnaire used is good. I still want your to be more to the point, What affects the hypothermia first then all numbers and text about the control group and intervention, as other reviewer tried to say in last review”.

 Response : We have rewritten the discussion in order of importance.

 3. Reviewer’s comment : “I feel you need to have a "closed loop" ref.in your summer of evidence section since this is a crucial part in communication especially when using videos as communication tools. (you need to mention this in the manuscript)”.

 Response : We have included closed loop reference in the summary of evidence section and under the need for study.

 Once again, we appreciate your valuable suggestions and feedback. Thank you.

# Reviewer report: Reviewer response for version 2

**DOI:** 10.5256/f1000research.173140.r332749

**Published:** 2024-10-24

**Authors:** Pracheth Raghuveer

**Affiliations:** 1 Department of Epidemiology, National Institute of Mental Health and Neurosciences, India, Bengaluru, Karnataka, India

The authors have made the required revisions. The article may now be accepted.

Is the work clearly and accurately presented and does it cite the current literature?

Yes

If applicable, is the statistical analysis and its interpretation appropriate?

Partly

Are all the source data underlying the results available to ensure full reproducibility?

Yes

Is the study design appropriate and is the work technically sound?

Partly

Are the conclusions drawn adequately supported by the results?

Yes

Are sufficient details of methods and analysis provided to allow replication by others?

Partly

Reviewer Expertise:

Mental Health, Non-Communicable Diseases, Health Systems Research

I confirm that I have read this submission and believe that I have an appropriate level of expertise to confirm that it is of an acceptable scientific standard.

# Reviewer report: Reviewer response for version 1

**DOI:** 10.5256/f1000research.169678.r316697

**Published:** 2024-10-01

**Authors:** Pracheth Raghuveer

**Affiliations:** 1 Department of Epidemiology, National Institute of Mental Health and Neurosciences, India, Bengaluru, Karnataka, India

I congratulate the authors for a timely study focusing on assessment of early neonatal practices to prevent neonatal hypothermia. However, there are a few reservations.

 1. Rationale or need for study is missing. The authors are advised to incorporate it.

 2. The methods need improvement. Instead of going for points, it is advisable to explain the methods in sentences. 

 3. In the results, I suggest it is better to present some of the key  factors associated with early neonatal practices.

 4. Discussion needs significant improvement. It is essential to compare the key findings with other studies, provide a balanced summary of the findings and highlight the strengths, novelty.

 5. Ethics section has appeared twice in the manuscript.

Is the work clearly and accurately presented and does it cite the current literature?

Yes

If applicable, is the statistical analysis and its interpretation appropriate?

Partly

Are all the source data underlying the results available to ensure full reproducibility?

Yes

Is the study design appropriate and is the work technically sound?

Partly

Are the conclusions drawn adequately supported by the results?

Yes

Are sufficient details of methods and analysis provided to allow replication by others?

Partly

Reviewer Expertise:

Mental Health, Non-Communicable Diseases, Health Systems Research

I confirm that I have read this submission and believe that I have an appropriate level of expertise to confirm that it is of an acceptable scientific standard, however I have significant reservations, as outlined above.

# Response

**Published:** 2024-10-07

**Authors:** Smrithi GM

Author Response 

 Smrithi GM

 Kasturba Medical College Mangalore,

 Manipal Academy of Higher Education,

 Karnataka,

 Manipal, 576 104,

 India.

 Dear reviewer,

 Thank you for taking time to review our article. We appreciate your feedback and would like to address your observation and inform you that the following changes have been done.

**1. Reviewer’s comment : “Rationale or need for study is missing. The authors are advised to incorporate it.”**

 Response : Rationale for need of study has been added after Summary of Evidence.

**2. Reviewer’s comment : “The methods need improvement. Instead of going for points, it is advisable to explain the methods in sentences.”**

 Response : We have revised and reviewed the article thoroughly and the methodology has been rewritten in the appropriate format as per your suggestion.

**3. Reviewer’s comment : “In the results, I suggest it is better to present some of the key  factors associated with early neonatal practices.”**

 Response : As per your suggestions, the results have also been rewritten to include only the key factors associated with early neonatal practices. The remaining points have been discussed under Discussion.

**4. Reviewer’s comment : “Discussion needs significant improvement. It is essential to compare the key findings with other studies, provide a balanced summary of the findings and highlight the strengths, novelty.”**

 Response : Discussion has been reviewed and updated. The key findings have been compared with other studies and the strengths have been highlighted in the critical reflection as per your suggestion.

**5. Reviewer’s comment : “Ethics section has appeared twice in the manuscript.”**

 Response :  We have removed the additional Ethics section which had been repeated in the manuscript.

 Once again, we appreciate your valuable feedback, and if you have any further comments or suggestions to improve the article, we will be happy to address them.

# Reviewer report: Reviewer response for version 1

**DOI:** 10.5256/f1000research.169678.r320822

**Published:** 2024-09-25

**Authors:** Linus Olson

**Affiliations:** 1 Karolinska Institutet, Solna, Sweden; 2 Department of Women's and Children's Health, Karolinska Institutet, Stockholm, Sweden

This is an interesting article.

 The article shows that we need to use interactive tools and videos as well as multiple training sessions.

 There are many things in results that are discussion material and needs to be moved down (As a reviewer, I don’t want to go into the details here since I would advise the authors that the whole manuscript should be rewritten).

 There are though some References discussing how communication is done in a closed loop way that should be added. (The technique of closed loop discussions go hand in hand with this manuscript so the authors have many to choose of and since there are culture differences in what you prefer using I choose not to give an example in this review report)

 I suggest to rewrite it in less statement in method and more as a scientific article. 

 The filming and video tools used is not described well enough. The type of camera, resolution and how they where used (blinded/masked for the group being filmed or not), how the videos were presented on a TV or a screen or in a bigger audience is not clear and what equipment used.

 All of this influence the learning concept for the mothers especially if they are in a crises or stress and since this is an important study that can have a big impact on how we teach and tell things it is of great importance for possible replications by others later.

Is the work clearly and accurately presented and does it cite the current literature?

Partly

If applicable, is the statistical analysis and its interpretation appropriate?

Partly

Are all the source data underlying the results available to ensure full reproducibility?

Yes

Is the study design appropriate and is the work technically sound?

Yes

Are the conclusions drawn adequately supported by the results?

Partly

Are sufficient details of methods and analysis provided to allow replication by others?

Yes

Reviewer Expertise:

Hyper- and hypothermia for neonates, Infections public health in LMIC countries, treatment with HT for HIE

I confirm that I have read this submission and believe that I have an appropriate level of expertise to state that I do not consider it to be of an acceptable scientific standard, for reasons outlined above.

# Response

**Published:** 2024-10-07

**Authors:** Smrithi GM

Author Response

 Smrithi GM

 Kasturba Medical College Mangalore,

 Manipal Academy of Higher Education,

 Karnataka,

 Manipal, 576 104,

 India

 Dear reviewer,

 We are very thankful for the availability and willingness of an international reviewer such as yourself to help us in peer review of the article titled “Assessment of early neonatal practices to prevent hypothermia ; A comparative study”.

 We have carefully considered your valuable feedback and have made efforts to improve the clarity and focus in our manuscript as per you suggestions and have made the changes accordingly.

 1. Reviewer’s Comment : “There are many things in results that are discussion material and needs to be moved down (As a reviewer, I don’t want to go into the details here since I would advise the authors that the whole manuscript should be rewritten).”

 Response : The format of the article has been re-written with the result section being made concise and discussion material shifted under appropriate heading.

 2. Reviewer’s Comment : “There are though some References discussing how communication is done in a closed loop way that should be added. (The technique of closed loop discussions go hand in hand with this manuscript so the authors have many to choose of and since there are culture differences in what you prefer using I choose not to give an example in this review report)”

 Response : We have addressed the methodology and explained in detail the materials used to communicate with and educate the mothers. In regards to communication, our video was made to enable a closed loop communication where we had clear objective of a set of points to educate the mothers. The information was clearly communicated in the language best understood and any questions asked following the video were addressed and followed up the next day prior to conducting the post intervention interview (ensuring closed loop communication).

 3. Reviewer’s Comment : “I suggest to rewrite it in less statement in method and more as a scientific article. 

 The filming and video tools used is not described well enough. The type of camera, resolution and how they where used (blinded/masked for the group being filmed or not), how the videos were presented on a TV or a screen or in a bigger audience is not clear and what equipment used.”

 Response : The methodology has been rewritten in less statement and more according to a scientific article. The key points like how the video was prepared, and used (as single blinded) and how it was presented have been elaborated in methodology.

 4. Reviewer’s Comment : “All of this influence the learning concept for the mothers especially if they are in a crises or stress and since this is an important study that can have a big impact on how we teach and tell things it is of great importance for possible replications by others later.”

 Response : Regarding the learning concept of the mothers, we have included only the mothers who have had an uneventful pregnancy and were of sound mind to participate in the study because we had to interview them pre and post video intervention. Any mothers who had any complications or seemed to be in stress due to pain or any other reasons had been excluded as they fulfil the exclusion criteria “inability of the mother to participate” of the study.

 Once again, we appreciate your valuable feedback, and if you have any further comments or suggestions to improve the article, we will be happy to address them.
